# Thresholds of Abnormality Perception in Facial Esthetics among Laypersons and Dental Professionals: Profile Esthetics

**DOI:** 10.1155/2020/2068961

**Published:** 2020-10-08

**Authors:** Raed H. Alrbata, Ayham Kh. Alfaqih, Mohammad R. Almhaidat, Ahmad M. Al-Tarawneh

**Affiliations:** Orthodontic Department, Royal Medical Services, Amman, Jordan

## Abstract

**Aim:**

To find thresholds at which laypersons and dental professionals from Jordanian population perceive abnormalities in sagittal positioning of upper and lower jaws as a major determinant to facial profile esthetics.

**Materials and Methods:**

Using photo editing software, a baseline profile image of a young male was manipulated on a 2 mm incremental basis to move each of the upper and lower jaws backward and forward relative to true vertical line (TVL) at which four variables of maxillary and mandibular retrusion and protrusion were researched. A total of 120 participants divided equally into four groups of laypersons, general dental practitioners (GDPs), orthodontists, and oral and maxillofacial surgeons (OMFSs) rated the images using an analog scale of 100 mm long. The image that showed the first statistical difference compared to the baseline was considered as a threshold of abnormality.

**Results:**

Laypersons, GDPs, and OMFSs perceived the abnormality in the maxillary retrusion at −5 mm to TVL, while orthodontists defined that at −3 mm. All dental professionals perceived the abnormality in the maxillary protrusion at +1 mm to TVL while the layperson group at +3 mm. A threshold of −7 mm mandibular retrusion to TVL was abnormally perceived by all groups. All dental professionals realized the abnormality in the mandibular protrusion at 0 mm to TVL while the laypersons at +2 mm.

**Conclusion:**

These thresholds regarding profile esthetics may contribute to the process of establishing proper orthodontic treatment planning that suits the highest facial esthetic standards.

## 1. Introduction

One of the major factors to consider for establishing the suitable orthodontic treatment for patients that matches their concerns about facial beauty and attractiveness is the profile. The majority of the clinical research studies concerning this issue agreed to the fact that facial profile beauty has changed over time [1, 2]. This was a serious motivation for orthodontists to keep updated with how different people perceive changes in the profile beauty so that the proper orthodontic treatment could be served and implemented successfully.

As the major treatment correction performed by the orthodontists target the area of lower face third, the relative positioning of the upper and lower jaws to each other, and to the whole face in the sagittal plane is of great importance in the profile establishment. However, some researchers reported a specific preference for straight profiles and bimaxillary retrusive profiles [3–6]; others found that the general public preferred the protrusive profiles over common cephalometric norms [7–9].

Nowadays, more focus is given specially by the orthodontists to the exact thresholds at which the upper and lower jaws are retrusively or protrusively perceived normally or abnormally by the general public and dental professionals who take part in the process of establishing the orthodontic treatment plan. Some researchers found that a threshold of 3 mm horizontal change of the mandibular or maxillary position was perceived negatively by orthodontists and laypersons [10]; others reported this at 2 mm for the orthodontists and oral and maxillofacial surgeons [11]. However, agreement between dental professionals and laypersons was found previously by some researchers concerning this issue [12–14]; others were against this and set different perception responses [15, 16]. However, the research concerning this issue is modest, and a comprehensive effort is needed to precisely define the thresholds at which different raters from a specific origin of professional and nonprofessional levels could perceive any deviation beyond normality concerning the sagittal positioning of both jaws as major constituents of the profile esthetics.

In this second part of the study, perception of the laypersons and dental professionals from Jordanian population to the profile esthetics will be investigated using multiple variables of the maxillary and mandibular retrusion and protrusion. The objective is to find the thresholds at which these raters could perceive abnormal positioning of both jaws in the sagittal plane.

## 2. Materials and Methods

The research protocol was approved by the Royal Medical Services human research ethics committee in November 2018, Amman, Jordan.

A standard profile image for a 19-year-old male was used for this part of the study. The subject selection procedure was based on the following criteria: age between 18 and 25 years, no previous orthodontic treatment or plastic surgery, and harmonious sagittal and vertical facial proportions in regard to the researched variables in the study as determined using cephalometric software (Viewbox version 4, dHAL Software, Kifissia, Greece). The profile image was taken for this subject in the natural head position with high standards of photography techniques using a DSLR camera (Nikon D3200, Lens 85 mm, and ring flash) after accepting and signing a consent form. It was then minimally modified to represent a baseline image with optimum vertical and anteroposterior profile standards of both jaws at 12.5% reduction compared to the original life-size image using photo editing software (Adobe Photoshop CS6 extended version 13.0, Adobe Systems Inc., CA, USA).

**Figure 1:**
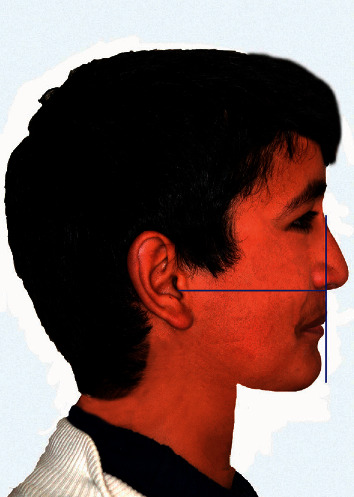
The baseline image used in the study as a reference for each variable with optimum projections of the major lower face profile points to the TVL (vertical and horizontal planes were removed for the rating process).

**Figure 2:**
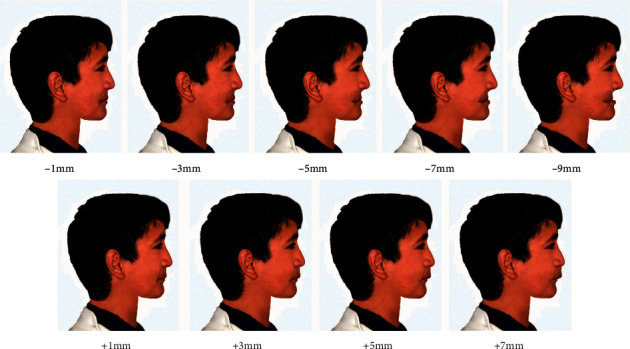
The manipulated profile images to affect maxillary retrusion and protrusion based on relation of the *A*^*∗*^ point to the TVL.

**Figure 3:**
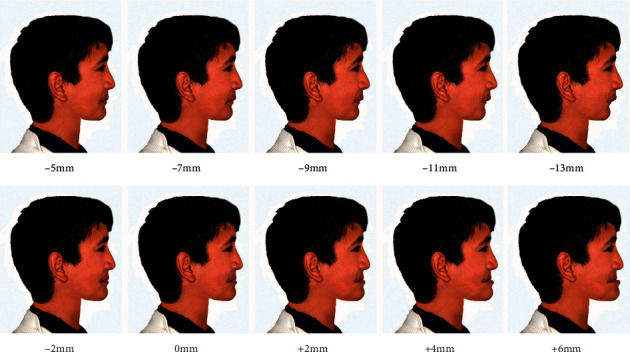
The manipulated profile images to affect mandibular retrusion and protrusion based on relation of the Pog^*∗*^ point to the TVL.

The reference line adopted in this study was the true vertical line (TVL) advocated by Arnett and McLaughlin as a line passing through subnasale and is perpendicular to the natural horizontal head position [17]. This line has contributed to the profile facial beauty by defining normal projections of different profile face points for males and females. The normal projections of the TVL to the major lower face profile points, subnasale, soft tissue *A* (*A*^*∗*^; −0.3 ± 1.0 mm), soft tissue *B* (*B*^*∗*^; −7.1 ± 1.6 mm), and soft tissue pogonion (Pog^*∗*^; –3.5 ± 1.8 mm) were set to the optimum values for a male face to represent the baseline image (Figure 1). This baseline image was then used as a template to generate multiple images based on manipulation of the anteroposterior positioning of the *A*^*∗*^ and Pog^*∗*^ points with the accompanied changes in the underlying upper and lower jaws, respectively. Four image variables were investigated: maxillary retrusion (*A*^*∗*^ from −1 to −9 mm to TVL with 2 mm interval), maxillary protrusion (*A*^*∗*^ from +1 to +7 mm to TVL with 2 mm interval), mandibular retrusion (Pog^*∗*^ from −5 to −13 mm to TVL with 2 mm interval), and mandibular protrusion (Pog^*∗*^ from −2 to +6 mm to TVL with 2 mm interval) (Figures 2 and 3).

In the maxillary protrusion variable, only 4 images were created and not 5 as for the other variables because the fifth image (+9 mm) was extremely unpleasant and unsuitable to present. The incremental manipulations were set based on the boundaries of the standard deviations of the normal projections for each point. The 2 mm incremental value was chosen for the purpose of precisely determining the thresholds at which first abnormality degree could be perceived by the raters while at the same time maintaining realistic facial changes. Each variable was rated separately. To avoid pattern detection, the baseline and the manipulated images for each of the four variables were given different symbols of 2 letters for each and randomly arranged at an A3 paper (one paper for each variable) in landscape orientation for evaluation at the same session to allow fair comparisons to be made. A duplicate of one of the images was used for each variable to assess intrarater reliability.

The raters participated in the study were the same laypersons, orthodontists, oral and maxillofacial surgeons (OMFSs), and general dental practitioners (GDPs) who participated in the part one of the study. The selection criteria for the laypersons were having a bachelor degree in any field except medicine or dentistry and no history of orthodontic or orthognathic surgery treatment. The GDPs have no higher education in any dental field. For orthodontists and OMFSs, they have experience of at least 3 years in their fields. The sample size was determined using a pilot study of similar selection criteria adopted in the study for each group. The layperson groups were considered as the reference to which other groups were compared to. The effect size was estimated at 0.95. On the basis of a significance level of alpha 0.05, the sample size was calculated to achieve 80% power and showed that 30 subjects for each group were necessary.

Each rater was given a booklet to fill in the esthetic perception values. Each booklet was having 4 pages with the manipulated images of the 4 profile facial variables (maxillary retrusion, maxillary protrusion, mandibular retrusion, and mandibular protrusion), a letter of appreciation for participation in the study, and a page of rating analog scale of 100 mm long (0, least attractive to 100, most attractive) with the symbols printed for each image at the end of each scale. The esthetic values were measured at the analog scales using a ruler by one orthodontist who was reasked after 2 weeks to repeat measurements for 10 raters from orthodontist group for all variables researched. This was to check for reliability of the measurements at which results of inter- and intraclass correlation coefficient were satisfactory with minimum of 0.88 and 0.87, respectively. All values were transferred to the excel file for the analyses needed.

### 2.1. Statistical Analysis

ANOVA and Tukey post hoc tests were performed to assess differences in the esthetic rating of all variables images compared to the baseline for each rater group. To determine intrarater agreement between the duplicate images used in the study and for the reliability test concerning the analog scale measurements, intra- and interclass correlation coefficients were used. All statistical tests were performed using statistical software SPSS v21 (IBM Corp., Armonk, NY, USA). The level of significance was set at *P* < 0.05.

## 3. Results

Table 1 shows the mean esthetic values of the four raters' groups for the baseline profile image along with the manipulated images of the maxillary retrusion and protrusion variables. All rater groups gave the highest esthetic perception values for the baseline image after which the mean values declined as the mm distance of the *A*^*∗*^ point was set more behind (for the maxillary retrusion images) or in front (for the maxillary protrusion images) relative to the TVL. The first statistical significance which appeared between the manipulated and baseline images was registered and considered as the threshold of abnormality perceived by the specific group. Laypersons, GDPs, and OMFSs perceived the abnormality in maxillary retrusion at −5 mm to the TVL, while orthodontists defined that at −3 mm. For the maxillary protrusion variable, all dental professionals perceived the abnormality at +1 mm to the TVL while the layperson group at +3 mm.

The results concerning the mandibular retrusion and protrusion variables are shown in Table 2. In the mandibular retrusion variable, the highest esthetic means for the layperson, GDP, and orthodontist groups were given to the −5 mm image, whereas the OMFSs assigned those to the baseline image. However, this was only statistically significant in the orthodontist group. For all groups, the −7 mm image was the threshold of abnormality perception as it showed statistical significance to both baseline and −5 mm image for the groups which gave this highest means.

The same scenario was shown in the mandibular protrusion images as the baseline image had lower esthetic means compared to the −2 mm image for all groups. Nevertheless, this was not statistically significant. All dental professionals perceived the abnormality in the mandibular protrusion at 0 mm to the TVL while the laypersons at +2 mm.

High levels of reliability were found between the duplicate images and between the baseline images which were separately rated for the four variables analyzed as all intraclass correlation coefficients were greater than 0.85.

## 4. Discussion

The use of TVL as a vertical reference plane for the diagnosis of dentofacial deformities and for corrective treatment planning was shown to be valid as reported in previous studies [18–20]. In this study, the baseline image was generated in reference to this line with the *A*^*∗*^ and Pog^*∗*^ points which were set to the optimum values to represent the sagittal positioning of the upper and lower jaws, respectively, after which the incremental manipulations for all images were performed without affecting the vertical height of the face or any other face parts such as the nose and forehead so that only anteroposterior positioning of the jaws was assessed excluding the impact of variations in other face parts such as the nose on the upper jaw or the submental area on the lower jaw. Although the *B*^*∗*^ point is the one that should represent the mandibular base, the Pog^*∗*^ point was considered instead to simplify the digital manipulations performed using software with the *B*^*∗*^ point kept in normal relation to the Pog^*∗*^ point with all manipulations performed.

A young Jordanian male subject was chosen, and his profile image was standardized for the purpose of the research methodology adopted in the study. For this, the resulted thresholds should comply well with the male standards of an Arab origin and not certainly for the females or even for another male with different age—due to different esthetic standards for some of the face variables—and ethnicity as normally this might be not similar. Moreover, equal number and homogenous age groups of males and females were considered for the raters. The reason beyond not investigating the impact of the gender and age factors for the subject and the raters is that to avoid ending up with a very large research and to pay more attention to the multiple variables which were researched at the same time in the study.

As shown in Table 1, the baseline maxillary positioning image of −0.3 mm *A*^*∗*^ point to the TVL representing a class I profile gave the highest esthetic means compared to the manipulated images for all the rater groups. This coincides well with the results of other studies which showed the class I orthognathic profile as more attractive than other profiles of abnormal sagittal positioning of the jaws [4–6]. As the incremental distance between the point and the line increased either in a forward or backward direction, the esthetic means were decreasing as reasonably expected for all groups.

In the maxillary retrusion variable, the orthodontist group was more sensitive in detecting the abnormality threshold at −3 mm to the TVL compared to the other groups at −5 mm. Such precise perception might be due to the fact that orthodontists, especially compared to the GDPs and OMFSs, used to give more attention to this variable as part of class III malocclusions and at earlier stages of growth at which growth modification could be implemented orthodontically. On the contrary, all dental professionals were able to detect the abnormality in the maxillary protrusion at +1 mm to the TVL compared to the laypersons who set this more forward at +3 mm.

For the mandibular positioning variable, except for the OMFS group rating mandibular retrusion images, the baseline image of −3.5 mm to the TVL showed lower esthetic means compared to the first manipulations performed at −5 and −2 mm for mandibular retrusion and protrusion, respectively. However, compared with the other groups, orthodontists showed a statistically significant preference of the −5mm image on top of the baseline image in the mandibular retrusion variable. As such 1.5 mm difference may not have that clinical significance; however, it gives impression of how orthodontists look more positively for mandibular retrusion to a limit compared to the optimum position relative to the TVL.

Interestingly, all groups perceived the threshold of abnormality in mandibular retrusion at −7 mm to the TVL. Meanwhile, the dental professionals groups were more sensitive in detecting the abnormality in the mandibular protrusion with the Pog^*∗*^ point in line with the TVL (0 mm) compared to the laypersons who pointed that out at +2 mm.

Laypersons were found in this study to favor a slight protrusion of the jaws compared to the dental professionals. This might be related to the feeling that such mild protrusion may give persons the appearance of “strong” jaws adding more positives to the personality attractiveness of human faces as perceived by the general public. Except for the maxillary retrusion variable, all dental professionals showed the same threshold of abnormality perception in all variables. This is in agreement with some studies that showed strong correlations between clinicians especially orthodontists and OMFSs in profile perception [3, 12].

Overall, the results of this study may coincide or contradict with other studies as multiple variables were investigated here. Some researchers found that laypersons were considered less sensitive than dental professionals in detecting abnormalities in horizontal changes of the profile esthetics [9, 15, 16]. This coincides with our results concerning the maxillary and mandibular protrusion variables but not to those of the retrusion variables of both jaws. However, the results of the latter variables agreed with the results of previous studies which showed agreement between lay judges and clinicians in the judgment of profile attractiveness [13, 14].

In fact, few researchers have focused at the exact thresholds at which different raters could perceive any abnormality in the sagittal positioning of the jaws. Barroso et al. found that laypersons may not be able to discriminate a 2 mm change in facial profile attractiveness [21]. Burcal et al. reported that orthodontists and OMFSs were more sensitive than laypersons to horizontal changes of 2 mm in the mandibular position [11]. Cochrane et al. found that both orthodontists and laypersons were relatively sensitive to 3 mm and more of horizontal changes in either jaw [9]. These results almost agreed to ours in a way or another. Fairly speaking, the incremental manipulations of the baseline images performed by the different researchers represent a crucial determinant to the concluded thresholds at the end.

A down-sized profile 2D image for one young male of an Arabs origin was considered as a baseline in this study. The impact of modifying the image size and dimension compared to the actual using another male or female subject of different age or ethnicity, facial soft tissue thickness, and male to female raters' differences might be needed to carefully consider such numerical esthetic perception. However, it should be stated that a systematic approach of rating different population samples to precise and small incremental manipulations of realistic images of different horizontal positioning of the maxillary and mandibular jaws standardized relative to a valid vertical line was followed comprehensively. Hopefully, these findings will contribute to the process of establishing proper orthodontic treatment planning that suits the highest facial profile esthetic standards as perceived by professional and nonprofessional persons.

## 5. Conclusions

Perception of abnormalities in the profile esthetics was researched in terms of the maxillary and mandibular jaw sagittal positioning variables relative to TVL (points *A*^*∗*^ for the maxilla and Pog^*∗*^ for the mandible). Considering the limitations encountered in this study as overall, different rater groups defined thresholds of abnormalities at which

- In the maxillary retrusion variable, orthodontists defined a threshold of −3 mm maxillary position to TVL at which profile esthetics were perceived as abnormal being more sensitive than laypersons, GDPs, and OMFSs who set that at −5 mm
- In the maxillary protrusion variable, orthodontists, GDPs, and OMFSs were able to detect the abnormality in the maxillary positioning at +1 mm to TVL compared to +3 mm for the laypersons
- For the mandibular retrusion, all groups perceived a threshold of −7 mm Pog^*∗*^ to TVL as abnormal mandibular retrusion
- In the mandibular protrusion variable, dental professionals were more sensitive to perception of the abnormality at which Pog^*∗*^ was located at the TVL compared to the laypersons who outlined that at 2 mm in front of the line

## Figures and Tables

**Table 1:** Means and SDs of the esthetic values for the baseline profile image and the manipulated maxillary retrusion and protrusion images as perceived by the four raters' groups.

Images	Laypersons (*N* = 30)			GDPs (*N* = 30)			Orthodontists (*N* = 30)			OMF surgeons (*N* = 30)		
	Mean	SD	Sig	Mean	SD	Sig	Mean	SD	Sig	Mean	SD	Sig
−9 mm	23.23	27.04	0.000^*∗*^	3.67	2.77	0.000^*∗*^	5.32	3.79	0.000^*∗*^	11.50	15.01	0.000^*∗*^
−7 mm	24.05	20.64	0.000^*∗*^	13.28	9.86	0.000^*∗*^	11.86	8.24	0.000^*∗*^	26.00	16.79	0.000^*∗*^
−5 mm	35.68	25.57	0.000^*∗∗*^	32.83	19.31	0.000^*∗∗*^	27.57	12.44	0.000^*∗*^	37.10	18.19	0.000^*∗∗*^
−3 mm	58.14	26.13	0.165	64.33	15.36	0.250	50.25	9.14	0.000^*∗∗*^	71.70	10.85	0.083
−1 mm	69.86	23.18	1.00	74.56	13.67	0.998	73.11	9.70	0.566	76.90	18.13	0.629
−0.3 mm (baseline)	71.18	22.30		77.61	17.80		77.48	11.37		84.73	7.19	
+1 mm	66.77	25.26	0.992	57.61	28.72	0.019^*∗∗*^	65.50	15.08	0.000^*∗∗*^	64.65	21.48	0.002^*∗∗*^
+3 mm	41.59	25.15	0.001^*∗∗*^	27.50	16.37	0.000^*∗*^	33.29	9.01	0.000^*∗*^	39.00	17.55	0.000^*∗*^
+5 mm	38.00	23.29	0.000^*∗*^	20.61	18.72	0.000^*∗*^	21.18	6.55	0.000^*∗*^	28.90	13.85	0.000^*∗*^
+7 mm	24.05	21.77	0.000^*∗*^	7.56	8.13	0.000^*∗*^	7.71	5.31	0.000^*∗*^	18.65	14.22	0.000^*∗*^

^*∗*^Significance at *P* < 0.05 as a result of Tukey post hoc test. ^*∗∗*^First significance appeared compared to the baseline image.

**Table 2:** Means and SDs of the esthetic values for the baseline profile image and the manipulated mandibular retrusion and protrusion images as perceived by the four raters' groups.

Images	Laypersons (*N* = 30)			GDPs (*N* = 30)			Orthodontists (*N* = 30)			OMF surgeons (*N* = 30)		
	Mean	SD	Sig	Mean	SD	Sig	Mean	SD	Sig	Mean	SD	Sig
−13 mm	27.32	21.57	0.000^*∗*^	11.50	12.74	0.000^*∗*^	7.46	5.70	0.000^*∗*^	29.70	16.21	0.000^*∗*^
−11 mm	23.73	17.58	0.000^*∗*^	15.83	11.04	0.000^*∗*^	12.43	7.94	0.000^*∗*^	22.50	15.87	0.000^*∗*^
−9 mm	41.41	26.04	0.000^*∗*^	35.89	21.33	0.000^*∗*^	29.43	8.72	0.000^*∗*^	44.05	13.91	0.000^*∗*^
−7 mm	44.73	23.51	0.006^*∗∗*^	52.44	29.32	0.001^*∗∗*^	43.96	11.99	0.000^*∗∗*^	41.95	17.12	0.000^*∗∗*^
−5 mm	73.77	21.37	0.996	82.28	12.72	0.983	79.71	12.11	0.000^*∗*^	72.15	24.19	0.890
−3.5 mm (baseline)	69.61	22.46		77.36	12.38		64.79	13.36		78.68	11.90	
2 mm	71.86	24.75	1.00	82.83	18.69	0.936	72.04	13.52	0.192	80.50	11.11	1.00
0 mm	59.41	27.24	0.825	62.72	19.89	0.010^*∗∗*^	42.75	17.42	0.000^*∗∗*^	56.35	18.70	0.000^*∗∗*^
+2 mm	33.00	21.75	0.000^*∗∗*^	29.78	17.50	0.000^*∗*^	24.68	8.90	0.000^*∗*^	32.70	12.78	0.000^*∗*^
+4 mm	30.00	24.32	0.000^*∗*^	15.39	10.68	0.000^*∗*^	11.07	7.50	0.000^*∗*^	26.60	15.00	0.000^*∗*^
+6 mm	23.64	25.10	0.000^*∗*^	6.22	9.56	0.000^*∗*^	5.61	3.84	0.000^*∗*^	13.50	13.20	0.000^*∗*^

^*∗*^Significance at *P* < 0.05 as a result of Tukey post hoc test. ^*∗∗*^First significance appeared compared to the baseline image.
